# Improving Compliance With Annual Structured Risk Assessment in an Older Adult Community Mental Health Team: A Two-Cycle Clinical Audit

**DOI:** 10.1192/bjo.2026.11721

**Published:** 2026-06-30

**Authors:** Muhammad Talha Farooq

**Affiliations:** Gartnavel Royal Hospital, Glasgow, United Kingdom

## Abstract

**Aims:**

Structured risk assessment is a core component of safe community mental health practice. Within NHS Greater Glasgow and Clyde, the Clinical Risk Assessment Framework for Teams (CRAFT) is used to document and review clinical risk for patients open to Community Mental Health Teams (CMHTs). Local guidance requires that CRAFT is updated at least annually, or sooner if risk changes. This audit aimed to assess compliance with annual CRAFT review within an elderly CMHT, identify factors contributing to non-compliance, and evaluate the impact of targeted interventions through a re-audit cycle.

**Methods:**

A retrospective two-cycle clinical audit was undertaken using electronic health records (EMIS). In cycle one, we reviewed all outpatients in medical clinics from 16 December 2024 to 14 March 2025, and nurse-led clinics from mid-February 2025 to mid-March 2025. We excluded patients who did not attend, inpatients, and those no longer open to the team. For each included patient, EMIS was searched for a CRAFT dated within the previous 12 months (i.e., dated March 2024 or later) and compliance was recorded as yes/no. Following cycle one, findings were presented and discussed in the community multidisciplinary team meeting, and an email was circulated to clinicians with updated guidance on CRAFT completion and method for identifying the date of the most recent CRAFT by searching with the keywords. Cycle two was completed three months later using medical clinics from mid-April to mid-July 2025, and nurse-led clinics from mid-June to mid-July 2025, applying the same inclusion/exclusion criteria and outcome measures.

**Results:**

In the first audit, 132 eligible outpatients were reviewed. 108/132 had a CRAFT updated within the preceding year, giving an overall compliance rate of 81.8%. There was 77.6 % (83/107) compliance within the medical team and 100% (25/25) within the nursing team. The plausible contributors to non-compliance were time pressure in medical clinics and the absence of automated prompts within EMIS. In cycle two, medical clinic compliance improved to 83.6% (102/122). Nurse-led clinics maintained a high compliance rate of 95% (38/40). Overall compliance increased to 86.4% in the re-audit.

**Conclusion:**

Compliance with annual CRAFT review in this elderly CMHT was high but not universal. A simple intervention, consisting of multidisciplinary feedback with practical guidance on screening for last CRAFT update, was associated with measurable improvement. Introducing electronic prompts and continuing periodic re-audit may help sustain gains and standardize accessible risk documentation across the team.

